# When Genetics Meet Hormones: Infertility and Normogonadotropic Hypogonadism From Robertsonian Translocation (14;21)

**DOI:** 10.7759/cureus.95189

**Published:** 2025-10-22

**Authors:** Hector R Gonzalez-Carranza, Nestor Saucedo-Conrado, Luis A Reyes-Vallejo

**Affiliations:** 1 Department of Urology, Hospital Angeles Metropolitano, Mexico City, MEX

**Keywords:** male hypogonadotropic hypogonadism, male infertility, rare genetic mutation, robertsonian translocation, spermatobioscopy

## Abstract

Infertility is a common issue among couples of reproductive age, and male patients may be affected by a variety of underlying conditions, with Robertsonian translocations representing a relatively rare cause. This case report presents a 46-year-old infertile man diagnosed with normogonadotropic hypogonadism caused by a balanced Robertsonian translocation 45, XY, t(14;21) (q10;q10). The patient reported unprotected sexual intercourse for a year and a half without achieving pregnancy. Physical examination revealed infantile body habitus, bilateral gynecomastia, scarce facial and pubic hair, decreased penile length, and atrophic testicles. Spermatobioscopy showed hypospermia and azoospermia, while testicular Doppler ultrasound revealed bilateral testicular atrophy. Hormonal profile indicated low serum total testosterone with low-normal follicle-stimulating hormone (FSH) and luteinizing hormone (LH) levels. Bone densitometry revealed osteopenia. Genetic analysis confirmed the presence of the Robertsonian translocation. This case highlights the importance of including cytogenetic analysis in the diagnostic workup of men with infertility, particularly those with azoospermia and hypogonadism. Robertsonian translocations can disrupt spermatogenesis and contribute to hormonal alterations, even in phenotypically normal patients. Early diagnosis facilitates appropriate counseling regarding reproductive options and management of hypogonadism to prevent metabolic complications and improve quality of life.

## Introduction

Infertility is defined by the World Health Organization (WHO) as the inability of a sexually active couple to achieve a spontaneous pregnancy within a 12-month period, in the absence of contraception. There are various causes of male infertility. Chromosomal abnormalities such as Robertsonian translocations represent a distinctive and underdiagnosed group. Robertsonian translocation consists of two acrocentric chromosomes, typically 13, 14, 15, 21, or 22, fusing their centromeric regions, resulting in a single balanced chromosome that preserves genetic material that may interfere with normal chromosomal segregation during meiosis. Infertility affects approximately 7% of men and is present in 10-15% of couples in reproductive age, with the male factor being the sole cause in 30-40% of cases [1,2].

Male hypogonadism accounts for 10.3% of the causes of infertility and can be defined as a clinical-biochemical syndrome characterized by low serum testosterone levels in the presence of symptoms. This disorder can be classified as primary or hypergonadotrophic in the context of a patient with primary testicular failure, low testosterone, and high follicle-stimulating hormone (FSH) and luteinizing hormone (LH) levels, or secondary or hypo gonadotrophic in the context of a patient with hypothalamic-pituitary-gonadal axis dysfunction, low testosterone, FSH, and LH levels [3].

The diagnostic approach to male infertility requires a systematic evaluation that integrates clinical, hormonal, genetic, and functional aspects. Initially, a clinical history, a detailed physical examination, testicular ultrasound, hormonal profile (testosterone, prolactin, FSH, and LH), and semen analysis are recommended.

Genetic evaluation has become increasingly important. Y Dong et al. studied 1,056 infertile men between the ages of 23 and 50 and reported that 16.1% had chromosomal abnormalities and 2.1% had chromosomal translocations. These included autosomal-autosomal reciprocal translocations, Robertsonian translocations, and autosomal-gonosomal reciprocal translocations. These balanced rearrangements are usually asymptomatic in carriers, who maintain normal somatic function but face reproductive challenges. In male patients, the pairing difficulties of the fused chromosomes during meiosis often result in spermatogenic failure and infertility [4].

Robertsonian translocations are the most common type of balanced translocations. Recent studies have shown that they can interfere with spermatogenesis through meiotic alterations that cause arrest in spermatocytic maturation, generating azoospermia or oligospermia, testicular dysfunction, and alterations of the hypothalamic-pituitary-gonadal axis, especially those involving chromosome 21 [5].

The present article presents the case of a 46-year-old infertile man who was diagnosed with normagonadotropic hypogonadism caused by Robertsonian translocation (14;21).

## Case presentation

A 46-year-old male presented to the urology and andrology clinic with a history of infertility, reporting regular unprotected sexual intercourse for the past year and a half without achieving conception. His family history was significant for a nephew diagnosed with Down syndrome, raising the possibility of an underlying genetic component.

The patient’s personal medical history included common childhood infections such as mumps and chickenpox. In adulthood, he had suffered from influenza four years earlier and a COVID-19 infection three years prior, the latter requiring hospitalization. No other chronic illnesses, surgeries, or exposure to gonadotoxic agents were reported.

Physical examination revealed a man standing 1.84 meters tall and weighing 110 kilograms, with an overall infantile body habitus and a gynecoid pattern of fat distribution. There was evident bilateral gynecomastia, and his secondary sexual characteristics were underdeveloped, as shown by sparse facial and pubic hair corresponding to Tanner stage II. Genital examination demonstrated a penis shorter than expected for his age and atrophic testes that were barely palpable. Additionally, his upper limbs appeared disproportionately long relative to his trunk, contributing to an overall eunuchoid appearance.

**Figure 1:**
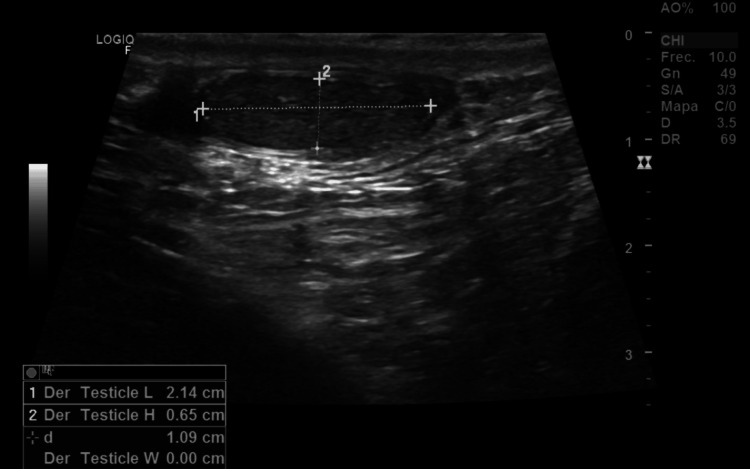
Testicular Doppler ultrasound showing reduced size of the right testicle Ultrasound image demonstrating the right testicle measuring 21 × 6 × 13 mm, indicative of testicular hypotrophy.

**Figure 2:**
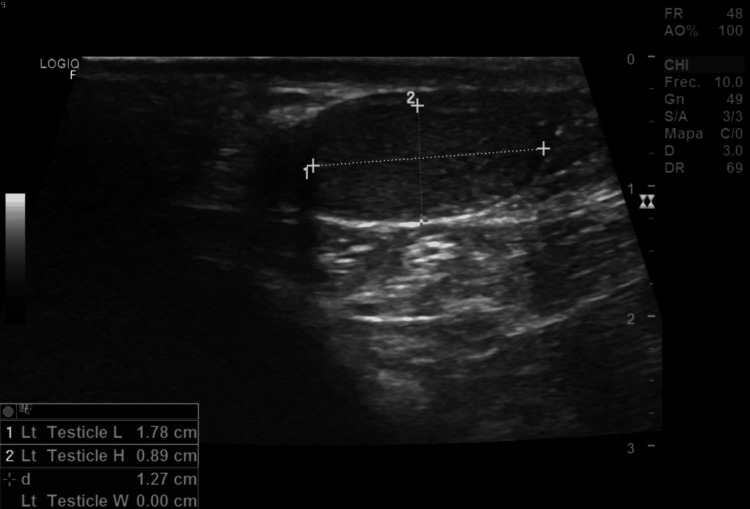
Testicular Doppler ultrasound demonstrating marked hypotrophy of the left testicle Ultrasound image showing the left testicle measuring 17 × 8 × 11 mm, consistent with significant testicular atrophy.

To further evaluate the cause of infertility, blood tests, a male hormonal profile, spermatobioscopy, and testicular Doppler ultrasound were ordered (Tables 1-2). The Doppler study revealed bilateral testicular atrophy with retractile testes, findings consistent with severe hypogonadism (Figures 1-2).

**Table 1:** Serum hormonal profile of the patient demonstrating low testosterone and gonadotropin levels

	Result	Reference range
Total testosterone	15.66 ng/dl	300-1000 ng/dl
Free testosterone	0.87 pg/dl	4.81-22.42 pg/dl
Follicle-stimulating hormone (FSH)	1.54 UI/ml	1.42-15.4 UI/ml
Luteinizing hormone (LH)	0.66 mUI/ml	1.5-9.3 mUI/ml
Prolactin	4.36 ng/ml	2.1-17.7 ng/ml

**Table 2:** Semen analysis demonstrating severe hypospermia and azoospermia Spermatobioscopy revealed a markedly reduced ejaculate volume of 0.1 mL with complete absence of spermatozoa, consistent with a diagnosis of hypospermia and azoospermia.

	Result	Reference range
Ejaculate volume	0.1 ml	>1.5 ml
Concentration	0	> 15.0 million
Motility	0 %	40 %

**Figure 3:**
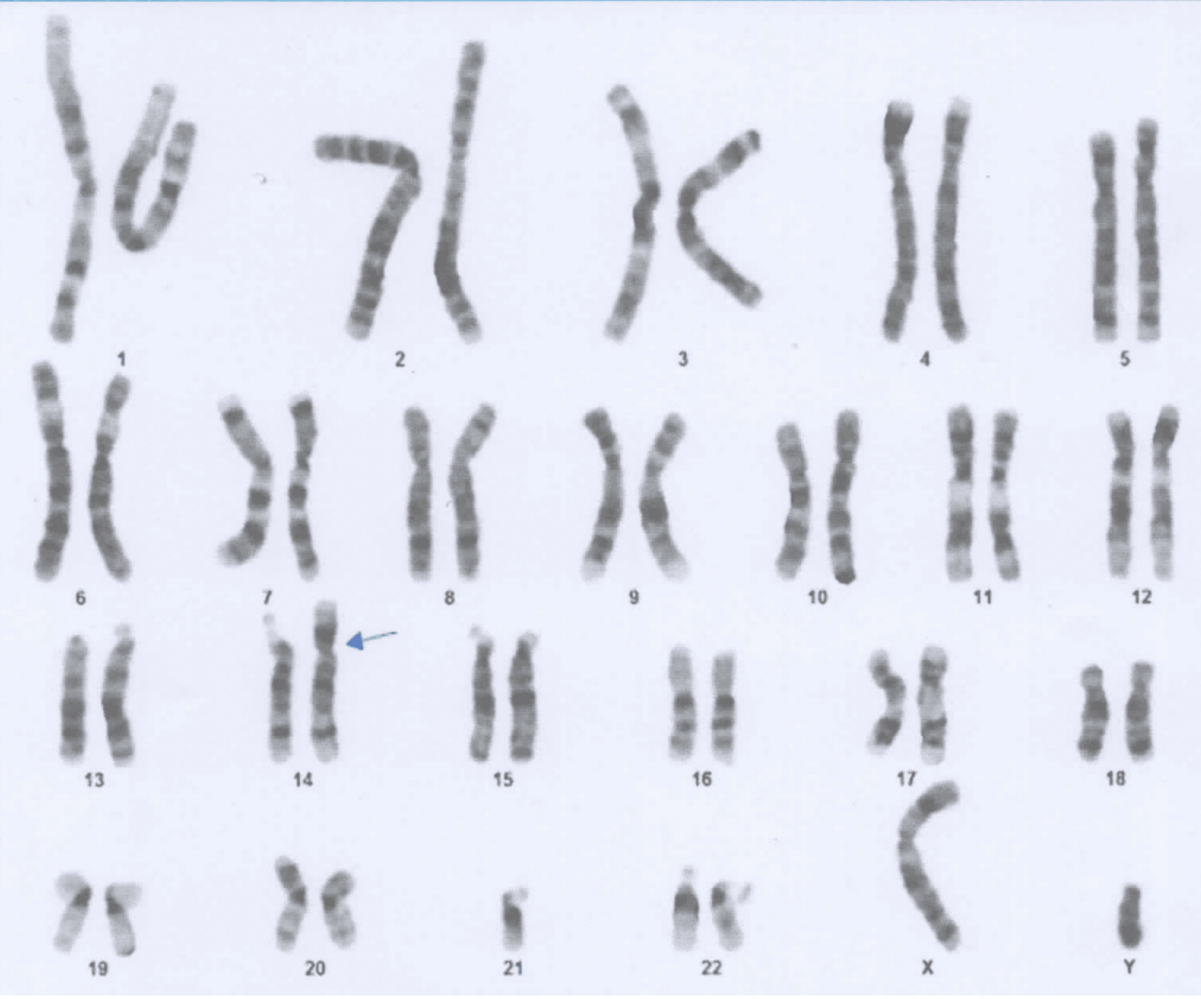
Cytogenetic analysis revealing a balanced Robertsonian translocation Karyotype result demonstrating a chromosomal rearrangement identified as 45, XY, t(14;21)(q10;q10), consistent with a balanced Robertsonian translocation between chromosomes 14 and 21.

Given the clinical suspicion of primary hypogonadism of genetic origin, a bone densitometry and karyotype analysis were requested to further characterize the condition. The densitometry revealed a lumbar bone mineral density (BMD) of 0.968 g/cm², with a T-score of -1.8, findings consistent with osteopenia. Genetic testing subsequently identified a structural chromosomal alteration corresponding to a balanced Robertsonian translocation: 45, XY, t(14;21)(q10;q10) (Figure 3).

Diagnosis of normogonadotrophic hypogonadism and balanced Robertsonian translocation 45, XY, t(14;21)(q10;q10) was established.

## Discussion

This case underscores the intricate relationship between male infertility and genetic abnormalities. Robertsonian translocations represent the most frequent type of structural chromosomal rearrangement, occurring in approximately one in 1,000 individuals in the general population [6]. These translocations usually involve acrocentric chromosomes, most commonly chromosomes 13 and 14, followed by 14 and 21. Carriers are typically phenotypically normal, as observed in this patient; however, reproductive outcomes may be compromised due to meiotic segregation abnormalities that lead to infertility [7].

Furthermore, the presence and severity of gynecomastia may have been exacerbated by the patient's obesity. A common mechanism seen in obese men with hypogonadism is the increase in peripheral aromatization of androgens to estrogens caused by adipose tissue. This results in a relative excess of estrogen and stimulates the development of breast tissue.

Infertility in such cases is primarily associated with meiotic disruptions resulting in spermatogenic arrest and azoospermia. Histological examination of the testes in patients with Robertsonian translocations often demonstrates seminiferous tubule hyalinization and germ cell aplasia, consistent with the testicular atrophy identified in our patient [8].

The finding of normogonadotropic hypogonadism, characterized by low total testosterone levels and low-to-normal gonadotropins, is particularly noteworthy [9]. Chromosomal rearrangements, including Robertsonian translocations, may alter the hypothalamic-pituitary-gonadal axis. In conjunction with primary testicular failure, this may result in insufficient gonadotropin elevation, contributing to the hormonal pattern seen in this case [10].

The patient’s osteopenia further supports the diagnosis of hypogonadism, as testosterone plays a crucial role in maintaining bone mineral density. This finding emphasizes the need for metabolic assessment and bone health monitoring in men with hypogonadal states [11].

Follow-up care should incorporate comprehensive genetic counseling for individuals with chromosomal abnormalities. Given this patient’s family history of Down syndrome in a nephew and his translocation involving chromosome 21, the case highlights the reproductive risks associated with Robertsonian translocation carriers [12]. Genetic counseling is therefore indispensable, particularly when assisted reproductive techniques are being considered.

This case also serves as a reminder for clinicians to consider genetic etiologies early in the diagnostic process. Atypical features, unexplained azoospermia, and hormonal abnormalities should prompt cytogenetic evaluation. Focusing exclusively on non-genetic causes could lead to prolonged and inconclusive workups. 

Managing infertility in men with Robertsonian translocations poses significant challenges. Assisted reproductive technologies (ART), such as testicular sperm extraction (TESE) in combination with intracytoplasmic sperm injection (ICSI), may provide a viable path to biological parenthood if sperm can be successfully retrieved. Preimplantation genetic testing (PGT) should be discussed as part of fertility planning to minimize the risk of transmitting chromosomal abnormalities and to enhance the likelihood of achieving a healthy pregnancy.

## Conclusions

This case emphasizes the importance of including cytogenetic analysis in the diagnostic workup of men with infertility, particularly those with azoospermia and hypogonadism. The identification of a Robertsonian translocation (14;21) in our patient highlights how chromosomal abnormalities can disrupt spermatogenesis and contribute to hormonal alterations, even in phenotypically normal patients. Early diagnosis facilitates appropriate counseling regarding reproductive options and management of hypogonadism to prevent metabolic complications associated with long-term testosterone deficiency, ultimately improving quality of life and informed reproductive decision-making.
